# Notices, &c.

**Published:** 1858-12

**Authors:** 


### NOTICES, &c.

*Quackery Unmasked*, by Dr. DANIEL KING, Boston, 8vo., 334 pp., $1, is the representative of conservative medicine, termed "old school," or "allopathy." It is suggestive to the masses, to all students of medicine, and to medical professors. To put down empiricism, our medical colleges must get a better material to work upon, and must make it their pride to excel in the quality of their graduates, rather than in their number. No reflecting man can read Dr. KING'S book without being largely supplied with facts for thought.

*Westminster Review*, for October, $3 a year, by LEONARD SCOTT and Co., 54 Gold Street, New York, contains nine articles, all written with scholarly ability. "Mr. NEWMAN and his Evangelical Critics," is an article of peculiar interest to Roman Catholics and Episcopalians. The *Edinburgh Review* is also received, having just entered its forty-ninth vol., $3 a year—Guizot's Memoirs, London Cotton Plant, &c., &c.

*The British and Foreign Medico-Chirurgical Review*, republished by the Messrs. WOOD, of New York, $3 a year, is the standard quarterly of legitimate medicine.

*The Medical and Surgical Reporter*, issued weekly, in Philadelphia, 8vo., $3 a year. It was founded eleven years ago. S. W. BUTLER, M.D., has long been identified with the *Reporter*, and is well known as the champion of honorable medicine. We heartily wish it a wide circulation and an extending usefulness.

For magazine monthly reading for 1859, ARTHUR, GODEY, GRAHAM, and PETERSON, of Philadelphia, are already preparing themselves for a fresh start, and are honorably striving to outvie each other in the interest and utility of their respective publications. Hereafter, *Graham* becomes the *American Monthly*.

HEALTH AND DISEASE; *a Book for the People*, by Dr. W. W. HALL, will be published on December 10th, by H. B. PRICE, No. 3 Everett House, New York. It will be sent, post paid, for one dollar. This book advises no medicine. It strongly discourages self-medication as pernicious, dangerous, and suicidal. The agencies it proposes for the maintenance of health and the cure of ordinary ailments, such as dyspepsias, neuralgias, costiveness, cold feet, and susceptibility to taking cold, are the judicious regulation of food, drink, air, exercise, cleanliness, and rest. In case these fail to give prompt and efficient relief, it advises that a physician of experience and education be called in, to originate all prescriptions, and to assume all the responsibility. As it answers no good purpose to make the people familiar with the symptoms of disease, this is avoided.

There is much that is entirely new to the masses in "*Health and Disease*," and not only new, but true and practical; and, if it meets with the welcome which our previous seven volumes have done, we shall be thankful, and feel besides a large indebtedness to a partial Public and Press.

*Young Men's Magazine*, monthly, $1.50 a year, edited by R. C. M'CORMICK, 348 Broadway, New York, is the only monthly in America devoted to the interests of young men, and is well worthy of their patronage. A sound, moral, and religious tone pervades every issue.

*Educational Journal*, Montgomery, Alabama, monthly, $1 a year, No. 2 (where is No. 1?), commends itself to the patronage of the South, and, if its subsequent numbers equal that for November, it will merit a liberal patronage.

Rev. W. A. SCOTT, D.D., of San Francisco, gives new proof of his industry and a facile pen in an eloquent and scholarly address on the national advantage of telegraphic intercourse with the Eastern Hemisphere.

FRANCIS & LUTTREL, Manufacturing Stationers and Printers, No. 45 Maiden Lane, New York, have prepared a *Daily Memorandum Book for* 1859, in a portable and handsome style, with several pages for miscellaneous memoranda, as also for a cash account for bills, payable and receivable. The paper is smooth, firm, white, and beautiful.

*The Pastor of the Old Stone Church; a Memorial of Rev. Ethan Osborne*, by the Messrs. MARTIEN, Philadelphia. The volume is most interesting, and beautifully executed, with an engraving of Mr. OSBORNE, who died in his hundredth year. He who would like to live that long would do well to purchase the book, and learn that, to live long, it is necessary, like the venerable clergyman, to be good and do good busily, "all the days of his appointed time, until his change come."

*The Tenant House; or, Embers from Poverty's Hearthstone*, by the Hon. A. J. H. DUGANNE, is published, in handsome style, by DE WITT, formerly DE WITT & DAVENPORT, 160 & 162 Nassau Street, New York; 490 pp., 12mo., sent free for $1.25.

Mr. DUGANNE was delegated to report to a committee of our State Legislature on the sanitary condition of the Tenant Houses of New York. This report was the result of a year's explorations among such buildings, some of which contain a thousand persons. The book was written after a careful examination of actual facts. The fidelity of the narration to observed facts, will strike any New Yorker, and, for ourselves, we hesitate not to say that, for terseness, for life-like description, and for power of language, we have never seen anything in Dickens to exceed the sketches of *The Tenant House*.

